# Simplified Roux-en-Y reconstruction after laparoscopic radical distal gastrectomy for gastric cancer

**DOI:** 10.3389/fsurg.2022.994659

**Published:** 2022-10-04

**Authors:** Yawei Qian, Guang Zhou, Feifei Chang, Xiaochun Ping, Guoliang Wang

**Affiliations:** ^1^ Department of General Surgery, The First Affiliated Hospital of Nanjing Medical University, Nanjing, China; ^2^ Division of Gastric Surgery, Department of General Surgery, The First Affiliated Hospital of Nanjing Medical University, Nanjing, China; ^3^ Department of General Surgery, Nanjing Central Hospital, Nanjing, China

**Keywords:** gastric cancer, distal, reconstruction, laparoscopic, modified

## Abstract

**Background:**

Although there were a variety of strategies for the alimentary tract reconstruction of patients with gastric cancer who underwent laparoscopic radical distal gastrectomy, it remains controversial regarding which procedure is optimal. We developed a simple technique for Roux-en-Y reconstruction during laparoscopic surgery and evaluated its technical feasibility and safety.

**Methods:**

Seventy-one cases of modified Roux-en-Y reconstructions after laparoscopic radical distal gastrectomy were consecutively performed in our hospital, from November 2020 to March 2022. A retrospective review of medical data was conducted. Intraoperative and postoperative outcomes, including operation time and incidence of postoperative complications, were collected and analyzed.

**Results:**

All procedures of laparoscopic distal gastrectomy with D2 lymph node dissection were successfully completed without any intraoperative complication. The mean number of retrieved lymph node was 38.8 ± 10.6. Mean operative time was 223.5 ± 42.4 min, whereas intraoperative blood loss was 102.2 ± 96.3 ml. No postoperative mortality was recorded. Six patients (8.5%) experienced postoperative complications and were managed conservatively. In addition, only two patients (2.8%) required rehospitalization during a median short-term follow-up period of 6 months.

**Conclusions:**

The modified method is a simple and safe approach for laparoscopic radical distal gastrectomy.

## Introduction

Gastric cancer (GC) is a highly malignant tumor with limited therapeutic effect. According to the latest statistics, over one million new cases of GC were diagnosed and about 769,000 deaths resulted from GC in 2020, making it the fifth most common cancer and the fourth most lethal cancer type worldwide (1). In the last few decades, great progress has been made in the treatment of GC, whereas radical surgical resection remains to be the only potential curative treatment option (2). For most of GC located in middle and lower stomach, particular in antrum and lesser curvature, distal gastrectomy (resection of the distal two-thirds of the stomach and anastomosis of the proximal stomach to the small bowel) is recommended (3).

Laparoscopic distal gastrectomy is gaining increasing popularity for early-stage GC with equivalent survival and faster recovery when compared with open surgery (4). Nevertheless, laparoscopic surgery is more technically demanding than open surgery, especially for alimentary tract reconstruction. Several studies showed that anastomosis-related complications are the most common complication subsequent to laparoscopic distal gastrectomy, including stomal stenosis and anastomotic leakage (5). In the context of laparoscopic distal gastrectomy, reconstruction of gastrointestinal tract is conducted in a limited working space with restricted vision, which may increase the risks of anastomosis-related complications. While various reconstruction methods after laparoscopic distal gastrectomy have been introduced, the preferred approach is still controversial. Here, we reported a simplified and safe technique for laparoscopic Roux-en-Y anastomosis and our early experience with this modified procedure.

## Patients and methods

### Patients

From November 2020 to March 2022, modified Roux-en-Y reconstructions after laparoscopic distal gastrectomy were performed for 71 consecutive patients treated for gastric cancer in the Department of Gastric Surgery at the First Affiliated Hospital of Nanjing Medical University. Their medical records and surgical notes were retrospectively collected and analyzed. Ethical approval for this study was granted by the Medical Ethics Committee of the First Affiliated Hospital of Nanjing Medical University, and informed consent from the retrospective patient cohort was waived.

### Surgical technique

The patient was placed in the supine reverse Trendelenburg position with legs apart and a carbon dioxide pneumoperitoneum (12–15 mmHg) was established through a subumbilical optical trocar under general anesthesia. A classic five-port method was used. Laparoscopic distal gastrectomy, accompanied by D1+ or D2 lymph node dissection, was performed according to the Japanese gastric cancer treatment guidelines 2018 (5th edition) (3). After the bulb of duodenum and the distal gastric body were transected using endoscopic linear stapler (Kangdi, Medtronic, China), the specimen was stored in the retrieving bag. Subsequently, a slightly modified Roux-en-Y technique was applied for alimentary tract reconstruction, as described below. All operations are performed by the same team of skilled surgeons.

### Roux-en-Y reconstruction with the modified technique

**Figure 1:**
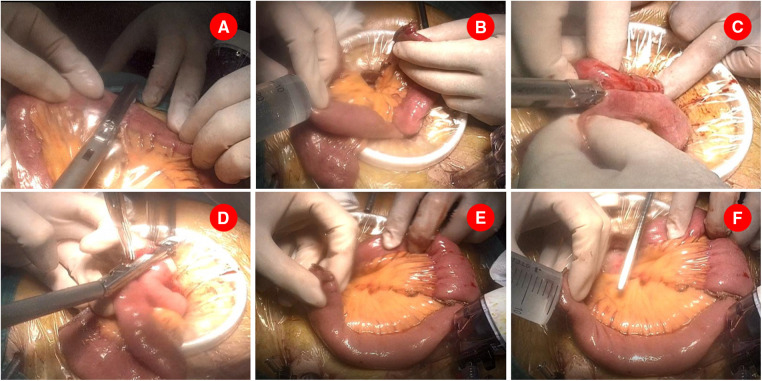
Jejunojenostomy was performed extracorporeally. (**A**). Jejunum was transected at 20 cm distal to the Treitz ligament. (**B**). Two small holes were made and an appropriate amount of saline was injected into each hole to facilitate the insertion of the stapler. (**C**). A 60 mm endoscopic linear stapler was separately inserted the above holes and created a side–side jejunojejunal anastomosis. (**D**). A 45 mm endoscopic linear stapler was used to close the common opening of enteroenterostomy. (**E**). A small opening was made at the stapling line of distal transected jejunum and a retracting suture was placed near the opening. (**F**). An appropriate amount of saline was injected into the opening.

A mini-laparotomy with a median length of 8 cm, depending on the size of specimen, was made on the upper abdomen, through which the specimen is retrieved and the surgical margins are confirmed. The ligament of Treitz was then identified and the jejunum was externalized *via* the mini-laparotomy. The jejunum was measured and transected at 20 cm distal to the Treitz ligament by using the 45-mm endoscopic linear stapler after its mesentery was divided to its root (Figure 1A). Jejunojejunostomy was performed extracorporeally. In particular, a small hole was made at the antimesenteric side of the proximal end of the transected jejunum, and another one was made on the antimesenteric side 40 cm away from the staple line of the distal transected jejunum. An appropriate amount of saline was injected into each hole to facilitate the insertion of the stapler (Figure 1B). The cartridge and anvil fork of the endoscopic linear stapler (60 mm, white cartridge) were separately inserted into each hole and fired to perform side–side jejunojejunal anastomosis (Figure 1C). As shown in Figure 1D, the created common entry of enteroenterostomy was then closed with the same linear stapler (45 mm, white cartridge) with cutting line parallel to the long axis of the intestine to avoid stenosis. As a result, the anastomotic stoma was established 15 cm away from the Treitz ligament and 30–32 cm away from the future gastrojejunostomy anastomosis. The mesenteric defect of the jejunojejunostomy was subsequently closed by interrupted sutures.

Before pneumoperitoneum was re-established, a small opening was made at the stapling line of distal transected jejunum on the antimesenteric side (Figure 1E). Similarly, an appropriate amount of saline was injected into the hole, and a retracting suture was placed near the opening, available for the assistant to pull the jejunum loop (Figure 1F). The operation proceeded back to laparoscopic procedure after returning the jejunum to the abdominal cavity. Accordingly, the surgeon moved to the right side of the patient and performed the following.

**Figure 2:**
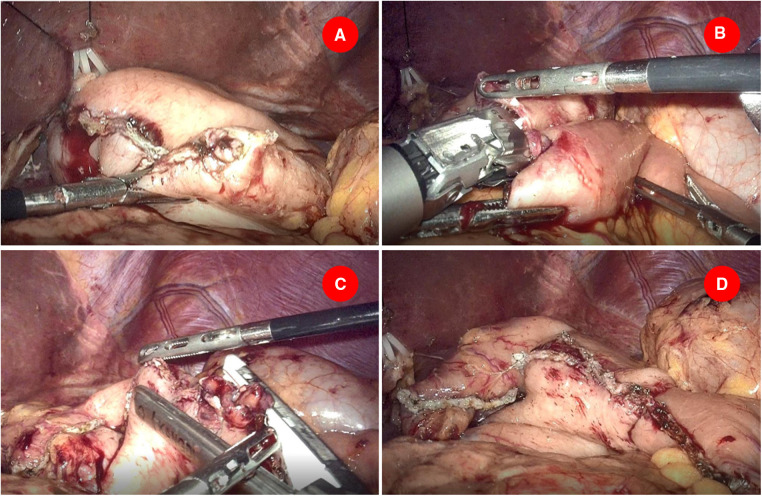
Gastrojejunostomy was performed intracorporeally. (**A**) Another small opening was made on the staple line of the greater curvature of the stomach stump. (**B**) A 60 mm endoscopic linear stapler cartridge was inserted to perform intracorporeal gastrojejunostomy. (**C**) Two 45-mm-long cartridges were employed to close the common entry of gastrojejunostomy. (**D**) The completion of the modified Roux-en-Y reconstruction.

Another small opening was made on the staple line of the greater curvature of the stomach stump and an appropriate amount of saline was injected into the remnant stomach (Figure 2A). A 60-mm-long endoscopic linear stapler with white cartridge was inserted from the right side of the patient to perform intracorporeal gastrojejunostomy. The anvil fork of the stapler was inserted into the jejunum and the stapler was partially closed and shifted toward the remnant stomach. The cartridge fork of the stapler was inserted into the stomach and the stapler is closed with the wall of the stomach and jejunum between its jaws, firing to create an antecolic afferent loop to greater curvature side-to-side gastrointestinal anastomosis (Figure 2B). Of particular note was that in order to avoid anastomotic tension, distal jejunum should be pulled cephalad before firing. Afterward, the camera was relocated into the right lower trocar and the endoscopic linear stapler was inserted through the trocar in the midline. The common entry site for gastrojejunostomy was closed by sequentially firing two 45-mm-long cartridges (Figure 2C). The completion of the modified Roux-en-Y reconstruction is shown in Figure 2D. Finally, Petersen’s defect was closed with nonabsorbable thread using continuous suture. Abdominal cavity drainage tubes were routinely placed near the gastrojejunal anastomosis and splenic fossa before abdominal closure.

## Results

In this study, it consisted of 52 males and 19 females with a median age of 59 years (range 30–87 years). The mean body mass index (BMI) was 23.83 ± 2.90 kg/m^2^. Tumor locations were mostly in the gastric antrum (*N* = 58). Two patients had a history of upper abdominal surgery. The detailed preoperative characteristics of these patients are summarized in Table 1. All 71 procedures of laparoscopic distal gastrectomy with D2 lymph node dissection were successfully completed without any intraoperative complication. No patient was required to convert to laparotomy, and no postoperative mortality was recorded. The intraoperative and postoperative parameters of this cohort are measured and summarized in Table 2.

**Table 1:** Demographics of 71 patients who underwent the modified Roux-en-Y reconstruction.

All patients	(*N* = 71)
Age (years)	58.87 ± 11.70
Sex	
* *Female	19
* *Male	52
BMI (kg/m^2^)	23.83 ± 2.90
ASA score	
* *I–II	64
* *III	7
NRS-2002 score	1.55 ± 0.96
Previous upper abdominal surgery	2
Previous ESD treatment	2
Location of tumor	
* *Middle	13
* *Lower	58
cTNM staging	
* *I–II	55
* *III–IV	16
Preoperative serum level of Alb (g/L)	37.55 ± 3.23
Preoperative serum level of HbA1c (%)	5.81 ± 0.91
Preoperative serum level of Hb (g/L)	129.34 ± 19.86

BMI, body mass index; ASA, American Society of Anesthesiologists; NRS-2002, nutrition risk screening-2002; ESD, endoscopic submucosal dissection; Alb, albumin; HbA1c, glycosylated hemoglobin, type A1C; Hb, hemoglobin.

**Table 2:** Operative parameters and short-term postoperative courses.

	(*N* = 71)
Incision length (cm)	7.1 ± 3.3
Operation time (min)	223.5 ± 42.4
Intraoperative blood loss (ml)	102.2 ± 96.3
Intraoperative complications	0
Postoperative complications	6
* *Anastomotic bleeding	1
Intra-abdominal bleeding	1
DGE	2
Chyle leakage	1
Duodenal stump external fistula	1
Postoperative mortality	0
Clavien–Dindo complications (grade III and above)	2
Time to first flatus	4.3 ± 1.1
Time to oral intake	6.5 ± 4.6
Postoperative hospital stay (days)	11.3 ± 5.2
pTNM staging	
* *I–II	59
* *III–IV	12
Differentiation	
* *Poorly	63
* *Moderately	8
Lymph node harvest (*n*)	38.8 ± 10.6
Number of cases requiring rehospitalization	2
Reasons for rehospitalization	
* *Intra-abdominal bleeding	1
* *Gastrointestinal anastomosis perforation	1

DGE, delayed gastric emptying.

Mean operative time was 223.5 ± 42.4 min, whereas estimated intraoperative blood loss was 102.2 ± 96.3 ml. The mean time of postoperative first flatus was 4.3 ± 1.1 days, and time of first oral intake was 6.5 ± 4.6 days. The mean postoperative hospital stay was 11.3 ± 5.2 days. No anastomotic leak was observed. Six patients experienced postoperative complications and were managed conservatively, including anastomotic bleeding (*N *= 1), intra-abdominal bleeding (*N *= 1), chyle leakage (*N *= 1), duodenal stump external fistula (*N *= 1), and delayed gastric emptying (*N *= 2). Postoperative pathology showed 63 cases of poorly differentiated adenocarcinoma and 8 cases of moderately differentiated adenocarcinoma. According to the TNM staging system, 59 cases were stage I–II and 12 cases were III–IV. All specimens were R0 resected and were confirmed margin negative. The mean number of retrieved lymph node was 38.8 ± 10.6.

**Figure 3:**
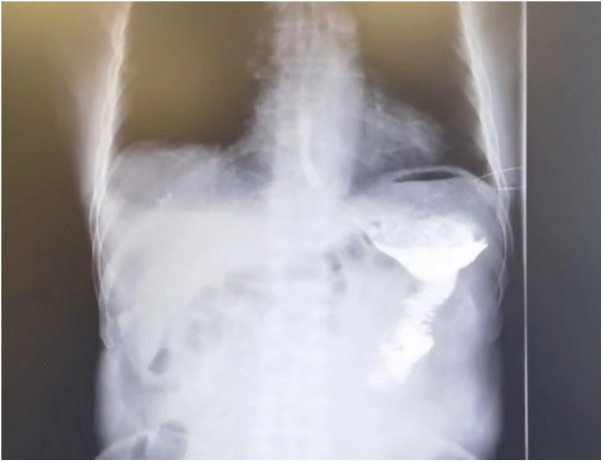
Six-month postoperative fluorography showing smooth passage of oral water-soluble contrast medium without anastomosis stenosis.

During a short-term follow-up period of 6 months (1–12 months), we did not observe any cases of recurrence or any cancer-related mortality (more than 30 days after surgery). Six-month postoperative checkup by contrast examination of the gastrointestinal tract (Figure 3) showed no anastomotic stenosis in our series. The Roux stasis syndrome occurred in nine patients and each case improved spontaneously in 1–2 weeks. These results indicated that our modified Roux-en-Y reconstruction is a safe and simple approach for laparoscopic distal gastrectomy.

## Discussion

Recent advances in surgical devices and techniques have allowed laparoscopic and even robotic-assisted gastrectomy and digestive tract reconstruction for the treatment of patients with gastric carcinoma. Since Kitano et al. first described a successful laparoscopy-assisted distal gastrectomy in 1994, laparoscopy distal gastrectomy is now considered a standard treatment option for early gastric cancer located in the middle or lower stomach (6). Long-term results from randomized controlled trials are awaited to support the evidence from cohort studies which have showed that, at least for early gastric cancer, laparoscopic resection is oncologically sound (7, 8). However, it is often difficult to perform the reconstruction of alimentary tract in limited and narrow operation space during laparoscopy, especially in patients with a small remnant stomach or in obese patients with thick abdominal walls. Even with continuous exploration of the best approach to reconstruct the digestive tract, there is still no consensus regarding which strategy is optimal for reconstruction during laparoscopic distal gastrectomy (9).

Similar to traditional laparotomy, the methods of digestive tract reconstruction after laparoscopic radical distal gastrectomy for gastric cancer mainly include Billroth-I, Billroth-II, and Roux-en-Y anastomosis. The Billroth-I operation is a technically simple type of reconstruction with preservation of physiological food passage, allowing food to pass through the duodenum and in which the remnant stomach was anastomosed directly to the duodenal stump (10). Nevertheless, high rates of remnant gastritis and esophagitis, even remnant gastric cancer or esophageal cancer, are observed in patients undergoing Billroth-I anastomosis (11, 12). In addition, it is difficult to perform Billroth-I reconstruction when the remnant stomach is too small or inappropriate for patients with advanced staging. The Billroth-II reconstruction is also a technically simple method with adequate excision range, which can help with the problem of anastomotic tension. However, Billroth-II reconstruction is rarely performed, especially in Japan, for the high incidence of bile reflux gastritis, which may be related to residual gastric cancer (13, 14). Even with Braun enteroenterostomy anastomosis, bile and pancreatic reflux into the gastrojejunal stoma is still inevitable in some patients (15).

The traditional Roux-en-Y gastrojejunostomy is initially created to reduce the risk of bile reflux by Roux in 1897, which, at the very least, anatomically solved the underlying problems of simple gastrojejunostomy plus jejunojejunostomy. Then, with the application of laparoscopy, Wittgrove et al. reported the first laparoscopic Roux-en-Y gastrojejunal anastomosis after gastrectomy (16). However, the Roux-en-Y procedure is technically complicated when it comes to laparoscopy (17). As with the traditional Roux-en-Y anastomosis, the laparoscopic approach is also possible to develop Roux stasis syndrome after operation, including chronic abdominal pain, hiccup vomiting, and postmeal fullness and other uncomfortable symptoms (18, 19). Therefore, laparoscopic Roux-en-Y anastomosis is not widely used and reported in laparoscopic radical gastrectomy for distal gastric cancer.

A new method called “uncut Roux-en-Y anastomosis” was proposed to prevent the incidence of Roux stasis syndrome by van Stiegmann and Goff in 1988 (20), and Uyama et al. reported the first laparoscopic-assisted uncut Roux-en-Y anastomosis in 2005 (21). Compared with traditional Roux-en-Y, the proximal jejunum has been closed without cutting off, which preserved the structural integrity of the small intestine and could theoretically reduce the occurrence of postoperative digestive tract transportation disorder and retention syndrome (22). In the operation, the proximal jejunum does not need to be transected and its mesentery does not need to be divided to its root, which can reduce the operation time and the incidence of bleeding (23). Nevertheless, the main problem with the uncut Roux-en-Y operation is that the stapled occlusion of the afferent loop is frequently recanalized, eventually allowing bile access to the gastric remnant (24).

The traditional Roux-en-Y reconstruction is more complex and time-consuming than other types of anastomosis. Recently, various modified laparoscopic Roux-en-Y reconstruction techniques have been attempted (25, 26). However, the best reconstruction for distal gastrectomy remains controversial. In this study, we reported a simplified method of Roux-en-Y reconstruction after laparoscopic distal gastrectomy. By the end of March 2022, we had performed Roux-en-Y reconstruction with a novel stapling technique after laparoscopic distal gastrectomy in 71 patients with gastric cancer. Encouragingly, only one patient developed an anastomotic complication related to bleeding. Over a median follow-up of 6 months, we observed that no patients died and only two patients required reoperation because of anastomotic perforation and abdominal bleeding. In addition, oral intake is easy and adequate after surgery because a sufficient sized anastomosis is obtained. We make the gastrointestinal anastomosis with a 60-mm linear stapler and close the common incision transversely in relation to the jejunal limb so that the size of the anastomosis is maximized. Even though the distal-side stapling line of the gastrojejunostomy is eliminated, the size of the anastomosis is sufficient. The closures of common entry hole by a linear stapler further make the stapling easy and secure. As our simplified operation is isoperistaltic stapled side-to-side anastomosis, closing the common opening at the top of the gastrointestinal anastomosis will not increase the risk of efferent loop stenosis.

Similar to other accounts in the literature, we have achieved satisfactory oncological outcomes with laparoscopic distal gastrectomy for early gastric cancer (27). All 71 patients underwent a D2 resection, which was adequate in achieving oncological clearance as suggested by the histology and follow-up. Our experience of this simplified operation is as follows: (i) when closing the common opening, the assistant should hold as little tissue as possible and ensure that whole layer of gastrointestinal wall was pulled, which is critical to prevent postoperative anastomotic stenosis; (ii) when closing the common opening of enteroenterostomy, the method of lateral closing should be adopted to ensure enough anastomosis; (iii) when performing intracorporeal gastrojejunostomy, the surgeon should move to the right side of the patient and the 60-mm-long endoscopic linear stapler insert from the periumbilical port to fire; (iv) when closing the common opening of gastrojejunostomy, two 45-mm-long cartridges, not 60-mm cartridges, were used and inserted from the right side 12-mm trocar port, which can make the operation more convenient. During this experience, we strived to establish our laparoscopic approach and improve on functional outcomes and quality of life after surgery.

However, this study is with limitations. More cartridges are used in this simplified Roux-en-Y reconstruction, and this is a retrospective study with small sample size that was concerned on the short-term outcomes of laparoscopic distal gastrectomy with modified Roux-en-Y anastomosis in a single center. A prospective cohort study or randomized control trial is necessary to assess the short-term and long-term outcomes of laparoscopic distal gastrectomy with modified Roux-en-Y anastomosis compared to others methods.

In conclusion, the development of our strategy for laparoscopic Roux-en-Y after distal gastrectomy is indicative of the trend toward intracorporeal reconstruction, which offers advantages in operation simplicity and avoidance of tension during cumbersome anastomosis. Our modified techniques for Roux-en-Y will increase in popularity and may represent the best option for reconstructions when appropriate surgical stapling instruments are used during operation.

## Data Availability

The original contributions presented in the study are included in the article/Supplementary Material, further inquiries can be directed to the corresponding author.
